# The importance of helplines in National Plans

**DOI:** 10.1186/1750-1172-9-S1-O12

**Published:** 2014-11-11

**Authors:** Monica Mazzucato, François Houyez, Paola Facchin

**Affiliations:** 1 Rare Diseases Coordinating Centre, Rare Diseases Helpline, Veneto Region, Italy; 2 European Organisation for Rare Diseases, EURORDIS

## 

In the last years, the Internet has attracted a considerable attention as a means to provide health-related information. Rare diseases patients have been described as active internet users, accessing the web both for searching activities and for communication purposes [1]. Despite the great opportunities offered both by health 1.0 and health 2.0 applications, some concerns arise as well, particularly regarding the following aspects: the accuracy and reliability of the information provided, a reported increase in feelings of anxiety and protection of personal information. Furthermore, the use of the internet in the health domain is linked to inequalities issues. E-health literacy [2] has been reported to be lower in patients and caregivers experiencing a high disease burden, as the one caused by many rare conditions. The analysis of the activities of 11 help-lines, operating in 7 European countries and part of the European Network of Rare Diseases Help-lines, outlines that the phone still represents a valuable source of information provision and support [3]. Although communication through e-mail can be easier to follow and more comfortable for the user when discussing sensitive issues, the phone conversation provides an immediate feedback and has a higher potential on reducing distress. The combined use of different tools, according to users’ needs and preferences, can reduce potential inequalities in access to appropriate information and support. The existence of help-lines providing information on rare diseases has been identified as one of the core-indicators for the evaluation of National Plans/strategies. The added value of setting up such services relies on different aspects. First, access. Whilst nearly seven out of ten EU households have an Internet connection (68%), 98% have a phone access, either through a fixed line or a mobile [4]. Furthermore, the divide between European countries is narrower considering phone access with respect to internet access. Second, whilst most of the health-related web contents are available in English, help-lines deliver information and support in local languages, reaching a potential higher number of persons. Phone lines have the possibility to adapt immediately to the knowledge and education level of the users for a more efficient communication. Finally, the public health value of help-lines should be underlined. They can produce visible results in the short term with limited costs and represent excellent observation tools through which the Health Authorities can receive, both from patients and from professionals, an immediate feedback on the functioning of the rare diseases policies put in place.
